# Aspirin plus dipyridamole has the highest surface under the cumulative ranking curves (SUCRA) values in terms of mortality, intracranial hemorrhage, and adverse event rate among 7 drug therapies in the treatment of cerebral infarction

**DOI:** 10.1097/MD.0000000000010123

**Published:** 2018-03-30

**Authors:** Jian-Jun Zhang, Xin Liu

**Affiliations:** Department of Pharmacy, Liaocheng People's Hospital, Liaocheng, P.R. China.

**Keywords:** cerebral infarction, drug therapy, efficacy, network meta-analysis, randomized controlled trials

## Abstract

**Background:**

The standardization for the clinical use of drug therapy for cerebral infarction (CI) has not yet determined in some aspects. In this paper, we discussed the efficacies of different drug therapies (aspirin, aspirin plus dipyridamole, aspirin plus clopidogrel, aspirin plus warfarin, cilostazol, warfarin, and ticlopidine) for CI.

**Methods:**

We searched databases of PubMed and Cochrane Library from the inception to April, 2017, randomized controlled trials (RCTs) met the inclusion and exclusion criteria were enrolled in this study. The network meta-analysis integrated evidences of direct and indirect comparisons to assess odd ratios (OR) and surface under the cumulative ranking curves (SUCRA) value.

**Results:**

Thirteen eligible RCTs including 7 drug therapies were included into this network meta-analysis. The network meta-analysis results showed that CI patients who received aspirin plus dipyridamole presented lower mortality when compared with those received aspirin plus clopidogrel (OR = 0.46, 95% CI = 0.18–0.99), indicating aspirin plus dipyridamole therapy had better efficacy for CI. As for intracranial hemorrhage (ICH), stroke recurrence, and adverse event (AE) rate, there were no significant differences of efficacy among 7 drug therapies. Besides, SUCRA values demonstrated that in the 7 drug therapies, aspirin plus dipyridamole therapy was more effective than others (mortality: 80.67%; ICH: 76.6%; AE rate: 90.2%).

**Conclusions:**

Our findings revealed that aspirin plus dipyridamole therapy might be the optimum one for patients with CI, which could help to improve the survival of CI patients.

## Abstract 2

Supplemental Digital Content is available in the text

## 1 Introduction

Cerebral infarction (CI), an ischemic stroke, is a frequent and serious complication of cerebral vascular disease, characterized by thrombosis, embolism, or systemic hemodynamic hypotension.^[1]^ It is accepted that CI is caused by atherosclerosis of large and small arteries, which results from an atherothrombotic or embolic blockage of the blood vessels supplying blood to the brain.^[2]^ Amongst stroke patients, 80% suffer from CI and 20% suffer from cerebral hemorrhage consequently. In addition, due to the rising number of patients, CI gradually is becoming a public health concern and is widely regarded as the first cause of disability and mortality.^[3,4]^ Generally, CI is known to be a multifactorial disease induced by complex interactions between environmental and genetic factors.^[5]^ Many intrinsic and extrinsic risk factors for CI have been established, such as diabetes, tobacco smoking, hypercholesterolemia, high blood pressure, and obesity.^[2]^ Currently, drug therapy is the main treatment for CI, including thrombolytic agents, anti-platelet aggregation drugs, anti-fibrinogen drug, anticoagulation, neuro-protective drugs, and other commonly used drugs. Since the anti-platelet aggregation drugs can prevent thrombosis, hence they have beneficial effects on prevention and treatment of CI.^[6–9]^

A previous study has shown that the application of anti-platelet aggregation drugs can reduce 11% to 15% of CI recurrence rate.^[10]^ At present, anti-platelet aggregation drugs include cyclooxygenase inhibitors (aspirin), ADP receptor antagonists (prasugrel, clopidogrel, ticlopidine), phosphodiesterase inhibitors (cilostazol, dipyridamole), platelet GP IIb/IIIa antagonists (abciximab), etc.^[11,12]^ Due to its protective abilities which result from a variety of different mechanisms, anti-platelet aggregation is being widely used to treat CI in recent years.^[10]^ And these widely used drugs had different efficacies in the treatment of CI. For example, a previous study showed that aspirin plus clopidogrel could significantly decrease vascular death compared with warfarin while non-significantly reduce the rate of recurrent stroke (including intracerebral hemorrhage), myocardial infarction, peripheral embolism in the treatment of ischemic stroke, and aortic arch plaques.^[13]^ Besides, it was found that aspirin combined with dipyridamole had better efficacy than aspirin alone in the treatment of CI.^[14]^ However, some findings suggested that warfarin in combination with aspirin had no additional benefits while increased the risk of adverse effects in comparison to aspirin alone.^[15]^ Therefore, when different effects of anti-platelet aggregation drugs were compared, it suggested clinical guidelines for drug treatment of CI.

Meta-analysis can compare the efficacy and safety of multiple interventions for the same disease, and select the best one based on interventions.^[16]^ Therefore, this study is designed to compare the efficacy of 7 drug therapies in the treatment of CI and provide more evidences for clinical application.

## 2 Materials and methods

### 2.1 Ethics statement

Our study is a meta-analysis and the ethics statement is not applicable.

### 2.2 Retrieval strategy

English databases, including PubMed and Cochrane library were used to retrieve relevant references in combination of manual retrieval. Retrieval range was from the establishment of the database up to January 2017. The search terms included drug therapy, aspirin, and cerebral infarction based on the combination of free words and key words.

### 2.3 Inclusion and exclusion criteria

Eligibility criteria: study types: randomized controlled studies; interventions: aspirin, aspirin plus dipyridamole, aspirin plus clopidogrel, aspirin plus warfarin, cilostazol, warfarin, and ticlopidine; study subjects: CI patients aged between 44 and 86 years; outcomes: intracranial hemorrhage (ICH), mortality, stroke recurrence, and adverse event rate (AE). Exclusion criteria: patients with severe artery occlusive diseases; patients allergic to clopidogrel, aspirin, or anticoagulant therapy; patients with advanced malignant tumor or dysfunction in blood, liver, and kidney; patients with severe hypertension; incomplete data; non-randomized controlled trials; overlap literatures; conference report, system evaluation, or abstract articles; non-English literatures.

### 2.4 Data extraction and quality assessment

Two researchers extracted the included literature data independently, according to the unified data collection form. Disputations during the process reached consensus through discussions of several investigators. The evaluation of randomized controlled trials was conducted by 2 or more researchers using Cochrane risk of bias assessment tool,^[17]^ which included 6 domains such as random assignment, allocation concealment, blinding of participants, incomplete outcome data, selective outcome reporting, and other sources of bias. The assessment of “yes,” “no,” or “unclear” was assigned to each domain for respective designation of a low, high, or unclear risk of bias. If “unclear” or “no” judgment was rated in any domain, the study was deemed as presenting a low risk of bias. If over 4 domains were assessed as “unclear” or “no,” a moderate risk of bias was designated to the study.^[18]^ Review Manager 5 conducted both quality evaluation and publication bias investigation (RevMan 5.2.3, Cochrane Collaboration, Oxford, UK).

### 2.5 Statistical analysis

Firstly, traditional pairwise meta-analyses were conducted for the studies with direct comparison of different treatment arms. Both odd ratios (ORs) and 95% credible intervals (CIs) estimations were pooled and reported. I-square test and Chi-square test were applied to test heterogeneity among different studies.^[19]^ Secondly, network diagrams were performed by the software R (V.3.2.1) package gemtc (V.0.6), and each node represented a single intervention, the size of node represented the sample size and the lines between the nodes represented the number of eligible studies. Thirdly, different interventions were compared with each other via Bayesian network meta-analyses, which were performed on the basis of non-informative priors for the purpose of effect sizes and precision. After 4 chains and a burn-in phase of 20,000 simulations, examinations confirmed convergence and lack of auto relation, hence conclusively producing direct probability statements deriving from another 50,000-simulation phase.^[20]^ In order to facilitate the process of the interpretation of ORs, the probability of each intervention was computed as the safest or most satisfactory cure method of Bayesian approach based on probability values, and thus summarized as surface under the cumulative ranking curve (SUCRA). A larger SUCRA value symbolized a better rank of intervention.^[21,22]^ All calculations were computed by R (V.3.2.1) package gemtc (V.0.6), and accompanied by Markov Chain Monte Carlo engine Open BUGS (V.3.4.0).

## 3 Results

### 3.1 In total, 16,771 participants from 12 two-arm RCTs and 1 three-arm RCT are selected in this study

**Figure 1:**
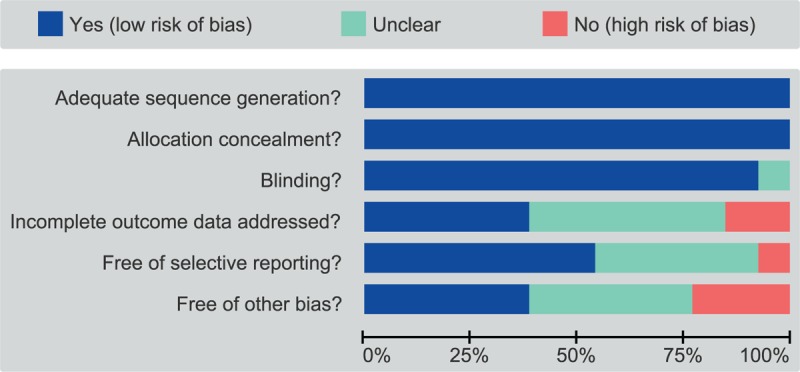
The Cochrane Collaboration's tool for the assessment of the risk of bias of the selected studies.

Initially, 1325 records were searched and 775 remained after exclusion of 10 duplicates, 151 letters or reviews, 226 non-human studies, and 163 non-English literatures. The full-text screening ruled out 198 non-cohort studies, 558 irrelevant trials, 6 with incomplete data, and 13 completely randomized controlled trials were comprised in this network meta-analysis^[23–35]^ (Supplementary Fig 1). A total of 16,771 CI patients were recorded in this study, with the most preferable treatment being aspirin and the least preferred being aspirin plus dipyridamole. The eligible studies were published between 2001 and 2017, of which subjects in 8 trials were Caucasians and the remainder was Asians. Twelve trials belonged to two-arm trials and 1 was in the category of a three-arm trial. The baseline characteristics are outlined in Table 1 and the Cochrane bias evaluation is shown in Fig. 1.

**Table 1:**
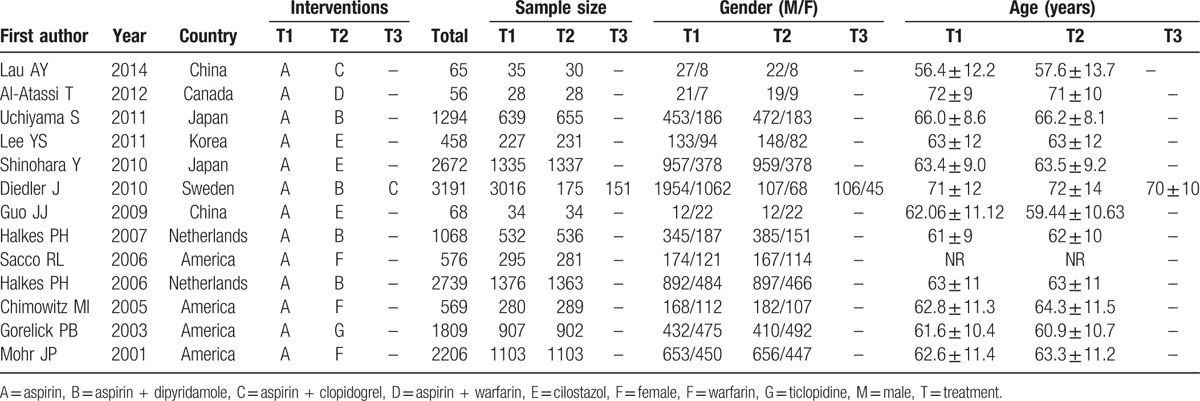
The baseline characteristics for included studies.

### 3.2 Results from pairwise meta-analysis of 7 drug therapies in the treatment of CI in terms of mortality rate, stroke recurrence rate, and AE rate

Pairwise comparisons upon 7 drug therapies revealed a lower mortality rate in patients who preferred aspirin and aspirin plus dipyridamole treatment than patients selecting treatment of aspirin plus clopidogrel (OR = 0.60, 95%CI = 0.40–0.90, OR = 0.31, 95%CI = 0.16–0.62, respectively). Aspirin had higher stroke recurrence rate as compared with cilostazol (OR = 1.46, 95%CI = 1.10–1.94), indicating aspirin had a worse efficacy in the treatment of CI. In comparison to aspirin plus clopidogrel and ticlopidine, aspirin also had higher AE rate (OR = 2.97, 95%CI = 1.99–4.43, OR = 2.26, 95%CI = 1.20–4.25, respectively). And the 5 drug treatments had no significant difference in terms of ICH (Table 2).

**Table 2:**
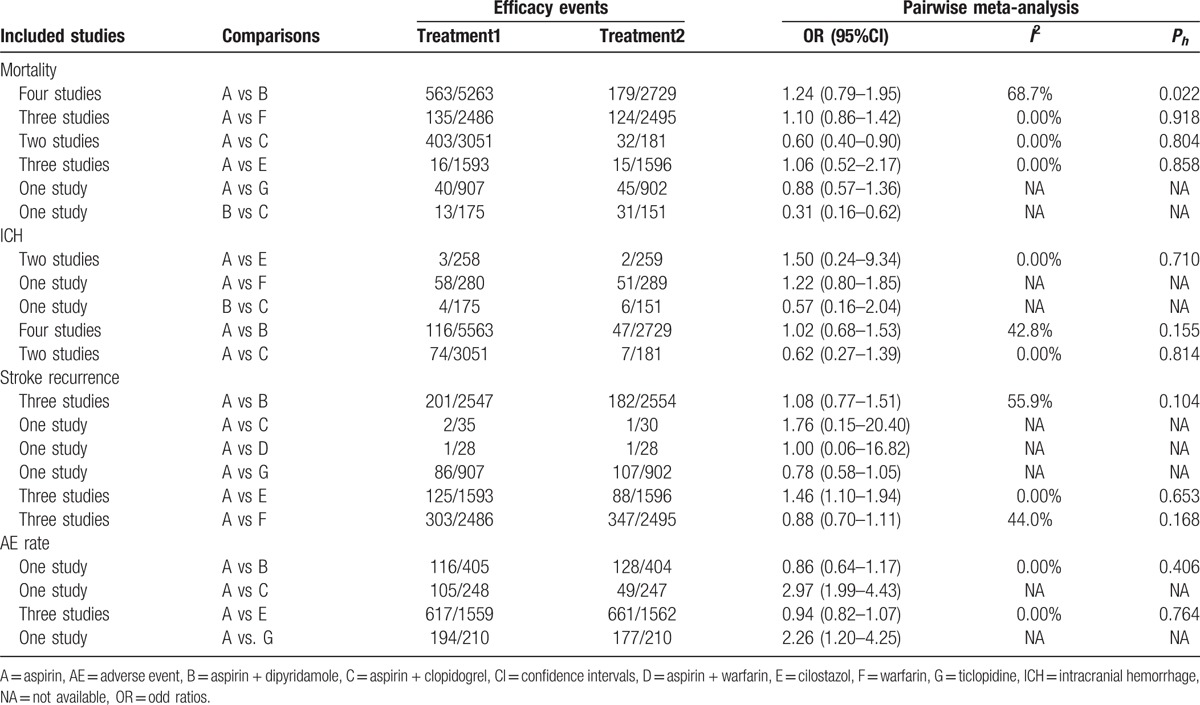
Estimated OR and 95% CI from pairwise meta-analysis for efficacy events in cerebral infarction patients.

### 3.3 Network evidence results showing more patients receive aspirin and less patients receive aspirin plus clopidogrel among 7 drug therapies in the treatment of CI

**Figure 2:**
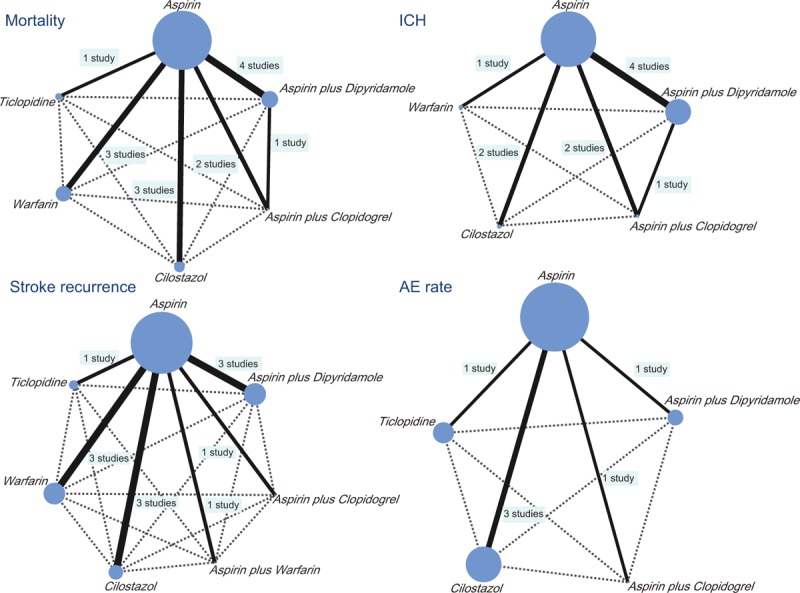
Network evidence graphs of mortality, ICH, stroke recurrence, and AE rate among 7 drug therapies in the treatment of CI. (Note: AE = adverse events, CI = confidence interval, ICH = intracranial hemorrhage).

This study included 7 drug therapies: aspirin, aspirin plus dipyridamole, aspirin plus clopidogrel, aspirin plus warfarin, cilostazol, warfarin, and ticlopidine. Among these drug therapies, the number of patients receiving aspirin was the highest, and aspirin plus clopidogrel was the least desired treatment (Fig. 2).

### 3.4 Results from network meta-analysis suggesting aspirin plus dipyridamole have better efficacy among 7 drug therapies in the treatment of CI

**Figure 3:**
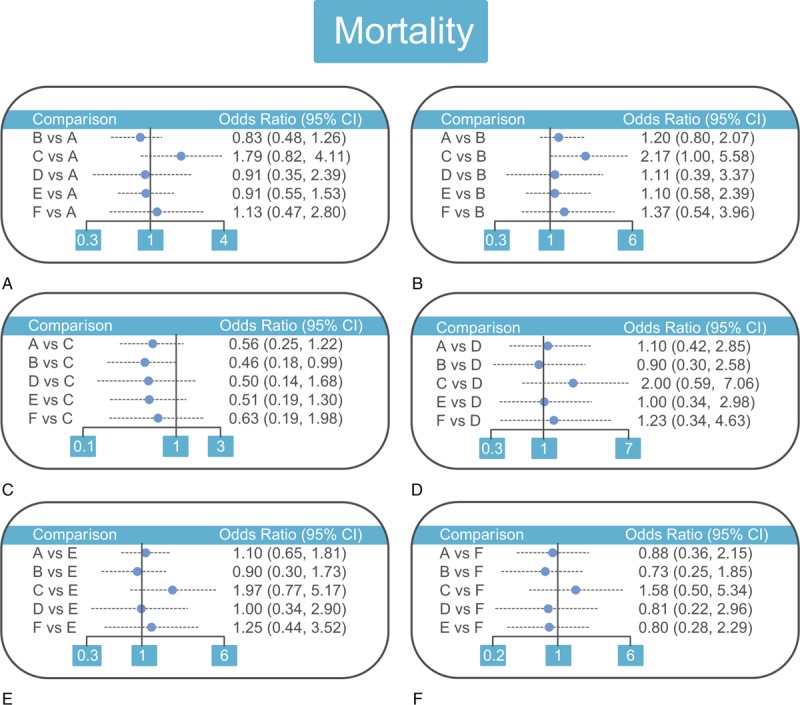
Relative relationship forest plots of mortality among (A) aspirin, (B) aspirin plus dipyridamole, (C) aspirin plus clopidogrel, (D) cilostazol, (E) warfarin, and (F) ticlopidine therapies in the treatment of CI. CI = confidence interval.

Between the 7 drug therapies, aspirin plus dipyridamole had lower mortality rate than aspirin plus clopidogrel (OR = 0.46, 95%CI = 0.18–0.99), suggesting that aspirin plus dipyridamole had better efficacy than aspirin plus clopidogrel in the treatment of CI (Table 3 and Fig. 3). ICH, stroke recurrence, and AE rate, all did not deliver any significant difference among the 7 drug therapies (Supplementary Fig. 2–4 and Supplementary Table 1).

**Table 3:**
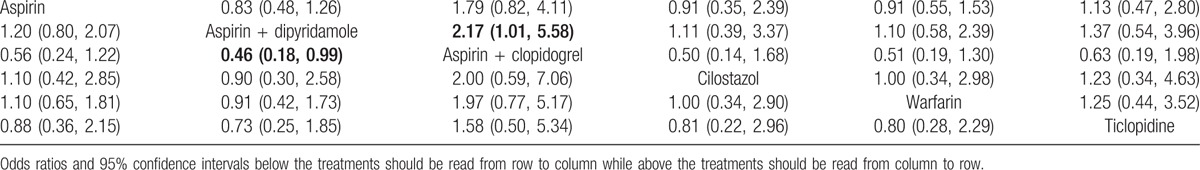
Odds ratios and 95% confidence intervals of six treatment modalities of mortality.

### 3.5 SUCRA values results indicating aspirin plus dipyridamole ranked the highest in terms of mortality, ICH, and AE rate among 7 drug therapies in the treatment of CI

**Figure 4:**
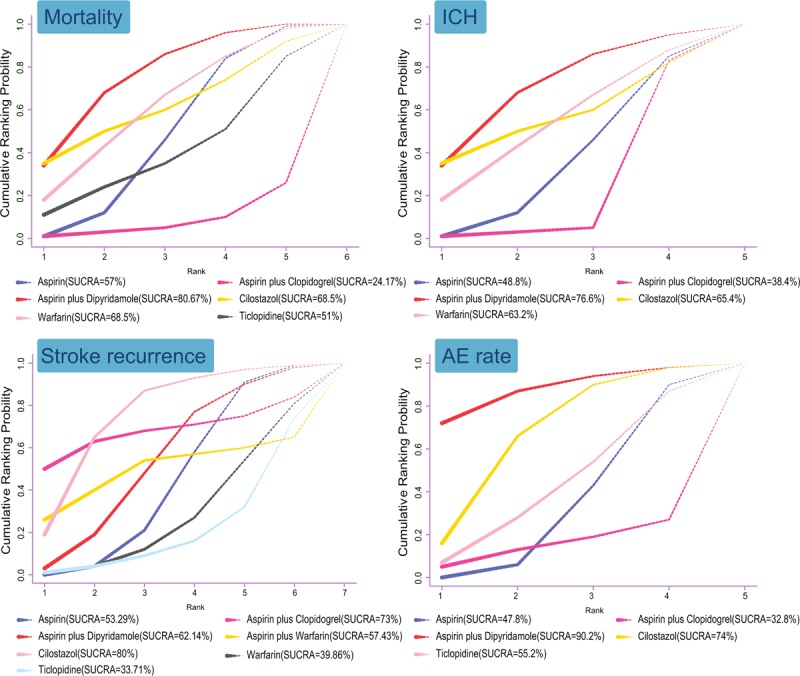
The SUCRA diagrams in terms of mortality, ICH, stroke recurrence, and AE rate among 7 drug therapies in the treatment of CI. (Note: AE = adverse events, CI = confidence interval, ICH = intracranial hemorrhage, SUCRA = surface under the cumulative ranking curves).

Figure 4 implied the rank probability of the treatment effect among all the therapies. The SUCRA value indicated that aspirin plus dipyridamole ranked the highest in terms of mortality, ICH and AE rate (mortality: 80.67%; ICH: 76.6%; AE rate: 90.2%), while concerning stroke recurrence. Cilostazol ranked the first (80.0%) and ticlopidine ranked the lowest (33.71%), which revealed that aspirin plus dipyridamole may be the best treatment regimens for CI patients, while the effect of aspirin plus clopidogrel on IC was the worst.

## 4 Discussion

This network meta-analysis evaluated the relative efficacy of 7 drug therapies (aspirin, aspirin plus dipyridamole, aspirin plus clopidogrel, aspirin plus warfarin, cilostazol, warfarin, and ticlopidine) on CI. According to the results, aspirin plus dipyridamole appeared to be the most effective treatment, while aspirin plus clopidogrel exhibited the poorest efficacy, correspondingly.

The study demonstrated that the combination of aspirin and dipyridamole was relatively the most effective in comparison to the other 6 drug therapies for CI. It is known that aspirin can achieve remarkable antiplatelet effects through acetylation of serine residue 530 of cyclooxygenase-1 (COX-1), causing irreversible inactivation of COX-1 and blocking the transformation of arachidonic acid into thromboxane A2 (a powerful platelet agonist and vasoconstrictor). Thus, aspirin can reduce risks of stroke and coronary artery disease.^[36,37]^ As the previous study indicated, dipyridamole was reported to reduce risk of vascular event after ischemic stroke similarly to aspirin.^[38]^ The combination of aspirin and dipyridamole played a more effectual role than aspirin monotherapy in ischemic stroke of presumed arterial origin,^[39]^ which indicated that aspirin and dipyridamole may exhibit synergistic action during the process. Also, aspirin plus extended-release dipyridamole presented a relative decreased risk of 22% in comparison to only aspirin.^[32]^ Besides, it was found that warfarin plus aspirin had superiority of reducing the risk of infarction and ischemic stroke while could lead to a higher risk of bleeding.^[40]^ However, Li et al^[41]^ found that aspirin plus dipyridamole therapy was efficacious in decreasing the recurrence of ischemic stroke without significant bleeding complication, which showed that aspirin plus dipyridamole was superior to warfarin plus aspirin treatment. In addition, aspirin plus dipyridamole had similar effects on preventing recurrent stroke when compared with clopidogrel.^[42]^

Additionally, our study also revealed that the combination of aspirin and clopidogrel was deemed as relatively the worst treatment method for CI. Clopidogrel is a derivative of thienopyridine that can inhibit platelet aggregation by selectively and irreversibly interdicting the adenosine diphosphate (ADP) receptor-P_2_Y_12_ on platelets, thus improving coronary syndromes.^[43]^ A previous study revealed that clopidogrel was superior to aspirin alone in reducing the risk of ischemic stroke.^[44]^ However, the combination of clopidogrel with aspirin failed to be more effective than clopidogrel monotherapy and instigated higher incidence of bleeding.^[45]^ Bhatt et al^[46]^ further proved this phenomenon by suggesting that the long-term addition of clopidogrel to aspirin was not available to broad population and may have harmed patients in primary prevention, which was also in line with the result of this study. It was noted that the two-step activation process of clopidogrel involved a series of cytochrome P450 enzyme metabolism which was susceptible to drug–drug interactions.^[47]^ It was conjectured that antagonistic action existed between aspirin and clopidogrel. Aspirin and clopidogrel resistance was recognized in the prevention and treatment of coronary artery disease.^[37,48]^ Besides, as compared with the combination of aspirin and a thienopyridine (ticlopidine or clopidogrel), aspirin alone was reported to be safer and more effective in the prevention of stent thrombosis after optimal intracoronary implantation of the CarboStent.^[49]^ A previous study also revealed that anticoagulation with warfarin may have better efficacy than aspirin plus clopidogrel as thromboprophylaxis in atrial fibrillation since aspirin plus clopidogrel could not decrease plasma indices of thrombogenesis and platelet activation.^[50]^

Network meta-analysis had some major advantages over traditional meta-analysis. It was not only a potent tool to integrate existing treatments for one clinical disease, but also served as an updated supplement to clinical guidelines and predicted future research needs.^[51]^ However, there were some limitations in this study. Firstly, this study was assessing outcomes only in European, American, and Asian population. Secondly, only 13 studies were enrolled in this meta-analysis and the number of included studies was relatively small with limited data and information. Therefore, it is hoped that these limitations will be improved in the future studies.

In summary, this study integrated and compared the efficacy of aspirin, aspirin plus dipyridamole, aspirin plus clopidogrel, aspirin plus warfarin, cilostazol, warfarin, and ticlopidine in the treatment of CI. The combination of aspirin plus dipyridamole treatment could achieve the greatest efficacy in comparison with other drug therapies, providing a significant guidance for the clinical treatment of CI.

## Author contribution

**Conceptualization:** X. Liu.

**Data curation:** X. Liu.

**Resources:** J.-J. Zhang.

**Supervision:** J.-J. Zhang.

**Writing – original draft:** J.-J. Zhang.

**Writing – review & editing:** X. Liu.

## Supplementary Material

Supplemental Digital Content
