# Supplementary material for: Aspirin plus dipyridamole has the highest surface under the cumulative ranking curves (SUCRA) values in terms of mortality, intracranial hemorrhage, and adverse event rate among 7 drug therapies in the treatment of cerebral infarction
Source: Medicine (Baltimore). 2018 Mar 30;97(13):e0123. doi: 10.1097/MD.0000000000010123 (PMC5895377; doi:10.1097/MD.0000000000010123)
Supplement: Supplemental Digital Content [file medi-97-e0123-s001.docx]

| Supplementary table 1: Odds ratios and 95% confidence intervals in the treatment of cerebral infarction in terms of ICH, Stroke recurrence and AE rate. | | | | | | |
| --- | --- | --- | --- | --- | --- | --- |
| **ICH** |  |  |  |  |  |  |
| **Aspirin** | 1.00 (0.57, 1.78) | 1.52 (0.40, 4.84) | 0.59 (0.07, 3.87) | 0.81 (0.32, 2.10) |  |  |
| 1.00 (0.56, 1.77) | **Aspirin + Dipyridamole** | 1.52 (0.46, 4.58) | 0.60 (0.06, 4.31) | 0.81 (0.28, 2.45) |  |  |
| 0.66 (0.21, 2.47) | 0.66 (0.22, 2.18) | **Aspirin + Clopidogrel** | 0.40 (0.03, 3.45) | 0.53 (0.14, 2.35) |  |  |
| 1.68 (0.26, 15.34) | 1.67 (0.23, 16.87) | 2.48 (0.29, 29.00) | **Cilostazol** | 1.38 (0.18, 14.83) |  |  |
| 1.23 (0.48, 3.12) | 1.23 (0.41, 3.62) | 1.87 (0.43, 7.39) | 0.73 (0.07, 5.64) | **Warfarin** |  |  |
| **Stroke recurrence** |  |  |  |  |  |  |
| **Aspirin** | 0.90 (0.64, 1.35) | 0.47 (0.02, 4.58) | 0.90 (0.02, 17.91) | 0.72 (0.43, 1.24) | 1.13 (0.77, 1.58) | 1.25 (0.70, 2.36) |
| 1.12 (0.74, 1.57) | **Aspirin + Dipyridamole** | 0.52 (0.02, 5.24) | 0.98 (0.02, 19.73) | 0.81 (0.40, 1.50) | 1.25 (0.70, 1.97) | 1.37 (0.67, 2.84) |
| 2.11 (0.22, 63.35) | 1.93 (0.19, 58.40) | **Aspirin + Clopidogrel** | 1.97 (0.03, 191.68) | 1.51 (0.16, 41.59) | 2.34 (0.23, 68.95) | 2.73 (0.26, 91.21) |
| 1.12 (0.06, 49.15) | 1.02 (0.05, 44.23) | 0.51 (0.01, 33.12) | **Aspirin + Warfarin** | 0.82 (0.04, 33.77) | 1.25 (0.06, 53.59) | 1.40 (0.06, 64.54) |
| 1.38 (0.81, 2.34) | 1.23 (0.67, 2.47) | 0.66 (0.02, 6.31) | 1.23 (0.03, 26.36) | **Cilostazol** | 1.55 (0.78, 2.88) | 1.72 (0.78, 3.96) |
| 0.89 (0.63, 1.30) | 0.80 (0.51, 1.43) | 0.43 (0.01, 4.28) | 0.80 (0.02, 16.62) | 0.65 (0.35, 1.28) | **Warfarin** | 1.11 (0.58, 2.34) |
| 0.80 (0.42, 1.43) | 0.73 (0.35, 1.50) | 0.37 (0.01, 3.92) | 0.72 (0.02, 15.73) | 0.58 (0.25, 1.28) | 0.90 (0.43, 1.73) | **Ticlopidine** |
| **AE rate** |  |  |  |  |  |  |
| **Aspirin** | 2.01 (0.74, 5.47) | 0.51 (0.09, 2.74) | 1.31 (0.82, 2.49) | 1.05 (0.47, 2.35) |  |  |
| 0.50 (0.18, 1.34) | **Aspirin + Dipyridamole** | 0.25 (0.03, 1.71) | 0.66 (0.23, 2.18) | 0.52 (0.15, 1.83) |  |  |
| 1.95 (0.36, 11.33) | 3.93 (0.59, 30.38) | **Aspirin + Clopidogrel** | 2.64 (0.47, 17.58) | 2.05 (0.34, 14.09) |  |  |
| 0.76 (0.40, 1.22) | 1.51 (0.46, 4.44) | 0.38 (0.06, 2.13) | **Cilostazol** | 0.80 (0.28, 1.95) |  |  |
| 0.95 (0.43, 2.14) | 1.94 (0.55, 6.83) | 0.49 (0.07, 2.98) | 1.25 (0.51, 3.52) | **Ticlopidine** |  |  |
| Notes: ICH = Intracranial hemorrhage;AE = adverse event. | | | | | | |

**Supplementary Fig 1.** Flow chart of participates in randomized controlled studies. In total, 16,771 participants from 12 two-arm RCTs and 1 three-arm RCT are selected in this study.

**Supplementary Fig 2.** Relative relationship forest plots of ICH among (A) Aspirin, (B) Aspirin + Dipyridamole, (C) Aspirin + Clopidogrel, (D) Cilostazol and (E) Warfarin therapies in the treatment of CI.

(Note: ICH = intracranial hemorrhage)

**Supplementary Fig 3.** Relative relationship forest plots of stroke recurrence among (A) Aspirin, (B) Aspirin + Dipyridamole, (C) Aspirin + Clopidogrel, (D) Aspirin + Warfarin, (E) Cilostazol, (F) Warfarin and (G) Ticlopidine therapies in the treatment of CI.

**Supplementary Fig 4.** Relative relationship forest plots of AE rate among (A) Aspirin, (B) Aspirin + Dipyridamole, (C) Aspirin + Clopidogrel, (D) Cilostazol and (E) Warfarin therapies in the treatment of CI.

(Note: AE = adverse events)
